# Implementation of safe infant sleep recommendations during night-time sleep in the first year of life in a German birth cohort

**DOI:** 10.1038/s41598-023-28008-1

**Published:** 2023-01-17

**Authors:** Vincent D. Gaertner, Sara Fill Malfertheiner, Janina Postpischil, Susanne Brandstetter, Birgit Seelbach-Göbel, Christian Apfelbacher, Michael Melter, Michael Kabesch, KUNO-Kids study group, Andreas Ambrosch, Petra A. Arndt, Andrea Baessler, Mark Berneburg, Stephan Böse-O’Reilly, Romuald Brunner, Wolfgang Buchalla, Sara Fill Malfertheiner, André Franke, Sebastian Häusler, Iris Heid, Stefanie Heinze, Wolfgang Högler, Sebastian Kerzel, Michael Koller, Michael Leitzmann, David Rothfuß, Wolfgang Rösch, Bianca Schaub, Stephan Weidinger, Sven Wellmann, Sebastian Kerzel

**Affiliations:** 1 Department of Pediatric Pneumology and Allergy, University Children’s Hospital Regensburg (KUNO) at the Hospital St. Hedwig of the Order of St. John, University of Regensburg, 93049 Regensburg, Germany; 2 Dr. von Hauner Children’s Hospital, University Hospital, Ludwig-Maximilians-University Munich, Munich, Germany; 3 University Department of Obstetrics and Gynecology at the Hospital St. Hedwig of the Order of St. John, University of Regensburg, Regensburg, Germany; 4 WECARE Research and Development Campus Regensburg at the Hospital St. Hedwig of the Order of St. John, Regensburg, Germany; 5 Institute of Social Medicine and Health Systems Research (ISMHSR), Otto von Guericke University Magdeburg, Magdeburg, Germany; 6 University Children’s Hospital Regensburg (KUNO), University of Regensburg, Regensburg, Germany; 7 Institute of Laboratory Medicine, Microbiology and Hygiene, Barmherzige Brüder Hospital, Regensburg, Germany; 8 ZNL Transfercenter of Neuroscience and Learning, University of Ulm, Ulm, Germany; 9 Department of Internal Medicine II, Regensburg University Medical Center, Regensburg, Germany; 10 Department of Dermatology, University Medical Centre Regensburg, Regensburg, Germany; 11 Clinic of Child and Adolescent Psychiatry, Psychosomatics and Psychotherapy, Bezirksklinikum Regensburg Medbo, Regensburg, Germany; 12 Department of Conservative Dentistry and Periodontology, University Hospital Regensburg, University of Regensburg, Regensburg, Germany; 13 Institute of Clinical Molecular Biology, Christian-Albrechts-University of Kiel, Kiel, Germany; 14 Department of Genetic Epidemiology, University of Regensburg, Regensburg, Germany; 15 Bavarian Health and Food Safety Authority (Landesamt für Gesundheit und Lebensmittelsicherheit) Munich, Munich, Germany; 16 Department of Pediatrics and Adolescent Medicine, Johannes Kepler University Linz, Linz, Austria; 17 Center for Clinical Studies, University Hospital Regensburg, Regensburg, Germany; 18 Department of Epidemiology and Preventive Medicine, University of Regensburg, Regensburg, Germany; 19 City of Regensburg, Coordinating Center for Early Interventions, Regensburg, Germany; 20 Department of Pediatric Urology, University Medical Center, Regensburg, Germany; 21 Department of Pediatrics, Dr. von Hauner Children’s Hospital, University Hospital Munich, Munich, Germany; 22 Department of Dermatology, Venereology and Allergy, University Hospital Schleswig-Holstein, Campus Kiel, Kiel, Germany; 23 Department of Neonatology, Hospital St. Hedwig of the Order of St. John, University Children’s Hospital Regensburg (KUNO), Regensburg, Germany

**Keywords:** Paediatric research, Risk factors

## Abstract

The aim of our study was to assess the extent to which families followed recommendations, issued by the German society for sleep medicine, for the prevention of sudden infant death syndrome (SIDS) during night-time sleep. Analyzing longitudinal data from a birth cohort located at the University Children’s Hospital Regensburg in Bavaria (Germany), we determined data regarding the infant's sleep location, sleep settings and body position, and exposure to environmental factors. Data were collected in a structured interview after birth and by standardized questionnaires at 4 weeks, 6 months, and 1 year of life, respectively. The majority of 1,400 surveyed infants (94% at 4 weeks) were reported to sleep in the parents’ sleeping room during the first months of life. While the most common furniture was a bedside sleeper (used by 48%), we also observed a considerable proportion of families who regularly practiced bed-sharing and, for 16% of infants, the parents’ bed was the default sleeping place. 12% of infants were still put regularly in the prone position. The vast majority (87%) of the infants were breastfed at some timepoint and 17% lived in a household with one or more smokers. Although most parents implemented many SIDS recommendations, our analysis illustrates a considerable gap between recommendations and intentions after birth on the one hand and actual implementation in real life on the other. The number-one deviation from the current SIDS guidelines during night-time sleep was bed-sharing with an adult.

## Introduction

The *sudden infant death syndrome* (SIDS) is still a leading cause of death during infancy in high-resource countries and hence a serious concern for most parents^1,2^. After identification of pivotal risk factors and modification of safe infant sleep recommendations^2–5^ to promote an exclusive supine sleep position for infants (i.e. face-up), room-sharing with the infant while avoiding bed-sharing with adults, usage of a firm sleeping surface, usage of a sleeping bag instead of a loose blanket, avoidance of overheating, and avoidance of nicotine exposure^1^, the SIDS incidence declined markedly in many countries in Europe and North America^6,7^. Some of these recommendations for a safe infant sleep environment do not exclusively target SIDS in its proper sense, but include other manifestations of sleep-related infant deaths, such as accidental suffocation and strangulation during unobserved sleep of the child.

In a previous paper, we determined the status quo of the knowledge and awareness of SIDS prevention measures among newborns’ mothers at the time of discharge from maternity ward^8^. We showed that the majority of mothers were able to actively report important recommendations for SIDS prevention and the overwhelming majority stated that they would never place their infant in the prone position for sleeping and that they do not plan to share the sleeping place with the infant nor to have loose items in the infant’s bed^8^. A multivariate regression analysis revealed no significant association between primipara status and knowledge of pivotal factors for SIDS prevention. In summary, immediately after their child’s birth the vast majority of mothers in our previous survey had good knowledge of the recommendations for a safe infant sleep, as mentioned in the corresponding national guideline^4^, and also the best intentions to implement these recommendations into practice. The aim of our present study was to assess the extent to which these intentions were actually followed in the infants’ first year of life and to determine which modifiable risk factors show the biggest gaps in the actual implementation during night-time sleep.

## Methods

### General design of the KUNO-Kids birth cohort

The KUNO-Kids study is a birth cohort that aims to assess multiple aspects of pediatric health and child development in a holistic approach^9^. As described in our previous publication Brandstetter et al.^9^, the study participants are pregnant women, who were recruited from our *University Department of Obstetrics* at the Hospital St. Hedwig (about 3,500 births/year)^9^. The hospital is located in Regensburg (Bavaria) in southeastern Germany and provides tertiary pediatric care for a total catchment area of approximately two million inhabitants.

The recruitment for the KUNO-kids birth cohort took place between June 2015 and April 2017, the data presented in this paper hence reflect the compliance with the SIDS guidelines at that time^5^. In the 2018 update of the corresponding guideline^4^, there have been no relevant changes regarding the parameters assessed in our present paper.

Written informed consent was provided by all study participants. Inclusion criteria were majority age and sufficient German language skills for informed consent. The sole exclusion criterion was an already ongoing participation of a sibling. The actual inclusion into the study occurred within the first 48 h after delivery^9^. The study was approved by the *Ethics Committee of the University Regensburg* (file number: 14-101-0347).

### Data collection on intentions regarding safe infant sleep after birth

As described in the publication outlining the general concept of our birth cohort^9^ and in a previous paper regarding maternal knowledge on SIDS recommendations^8^, published by the same author group as the present paper, we obtained the data on the infant's intended sleep environment and the intentions to implement SIDS recommendations during a standardized and structured interview, which took place at the maternity ward between 24 and 72 h after birth of the newborn child. In correspondence with our previous study^8^ we asked the mothers the following specific questions (prompted). To maintain comparability, the same wording as in our previous paper Fill Malfertheiner et al. 2022^8^ was used.

- “*In which room is the infant going to sleep?*” Possible answers: (i) in the parents’ sleeping room, (ii) in his/her own nursery, (iii) in a children’s room together with a sibling, (iv) in another room, (v) don’t know yet, (vi) not specified.
- “*Where are you going to put your infant to sleep?*” Possible answers: (i) own crib, (ii) parents’ bed, (iii) (single side-open) bedside sleeper, (iv) classical cradle/bassinet, (v) don’t know yet, (vi) not specified.
- “*Do you plan to put the child in a prone position for sleeping, thus face down?*” Possible answers: (i) always (100% of the time), (ii) mostly (75%), (iii) often (50%), (iv) rarely (25%), (v) never (0%), (vi) don’t know yet, (vii) not specified.
- *“Which of the following objects are you going to give the infant into the bed for sleeping?*” Possible answers for multiple choice: blanket, sleeping bag, classical pillow, breastfeeding/nursing pillow, burp cloth, baby hat, gloves, baby nest, pacifier, cuddly toy, music box, or none of these, don’t know, not specified, respectively.
- *“Which room temperature do you aim for in the room in which your child is going to sleep?”* Possible answers: (i) below 16 °C, (ii) 16–18 °C, (iii) 18–20 °C, (iv) 20–22 °C, (v) above 22 °C, (vi) don’t know, (vii) not specified.
- *“Are you going to breastfeed the infant?”* Possible answers: (i) yes, (ii) no, (iii) don’t know, (iv) not specified.
- *“How many persons regularly smoke in your household?”* Possible answers: (i) nobody, (ii) 1 person, (iii) 2 persons, (iv) 3 persons, (v) 4 or more persons, (vi) don’t know, (vii) not specified.

The mothers in our study did not receive any education regarding SIDS recommendations from the study team or other hospital staff prior to answering these questions. Structured counseling on this topic is a mandatory part of the 2nd well-child visit with the pediatrician, which is normally performed between the 3rd and 10th day of life in Germany. Therefore, almost all mothers in our study received counseling on healthy infant sleep immediately after the study interview by pediatric medical staff on the maternity ward. However, this counseling was not part of our study. Independently, mothers in our study collective who already had an older child might not have been naïve regarding information about SIDS prevention.

### Data collection on implementation of safe infant sleep recommendations during first year of life

Families received standardized questionnaires via mail at the following points of time during first year of life (related to the child's age): at one month, at six months, and shortly before the first birthday. The parents were asked the corresponding questions as in the interview after birth (see above) with the difference that in the questionnaires the questions were grammatically rephrased related to the current situation:

- “*In which room does your infant usually sleep at night?*” (Default sleeping location at which the infant usually spents most of the sleeping time)
- “*Wherein do you put your infant usually to sleep?*” (Default sleeping furniture in which the infant usually spents most of the sleeping time)
- “*How often do you put the infant in a prone position for sleeping, thus face down?*”
- *“Which of the following objects do you give the infant into the bed for sleeping?*”
- *“What is the temperature in the room in which your child usually sleeps?”*
- *“How many persons regularly smoke in your household?”*

The possible answers were identical to the corresponding questions in the interview after birth (see above).

In order not to exclude children receiving expressed breast milk, the question about breastfeeding was rephrased to “*Does your child currently still receive breast milk?*”. Additionally, for a better differentiation of bed-sharing, the parents were asked to quantify the usual time of bed-sharing:

- “*How often does your infant usually co-sleep with an adult in one bed at the same time?*” Possible answers: (i) always (on 100% of the nights), (ii) often (on more than 50%), (iii) rarely (on less than 50%), (iv) never (0%), (v) don’t know, (vi) not specified.

### Statistical analysis

By means of descriptive statistics, the knowledge and actual implementation of the SIDS prevention measures are presented as relative and absolute frequencies. Only children (and their families) with a complete data set at all four time points (birth, 4 weeks, 6 months, and 1 year) were included in the current analysis for this paper. Unanswered individual questions were categorized as *missing data*. To determine an interrelation of pivotal risk and protective factors, we performed an association analysis using a Chi-square test. All statistical analyses were performed with R statistics, version 4.2.1^10^. Figures were plotted with GraphPad Prism ® 6.07 (La Jolla, USA).

### Ethics approval

This study was performed in line with the principles of the Declaration of Helsinki. Approval was granted by the Ethics Committee of the University Regensburg (file number: 14-101-0347).

### Consent to participate

Written informed consent was obtained from all parents.

## Results

### Characteristics of sample

Population characteristics are outlined in Table 1.

**Table 1:** Characteristics of the study population.

	Study participants (*n* = 1,400)			Dispersion
	Number	Percentage	Value	
Mothers				
Age (years) (Mean ± SD)			32.6	(4.1)
Migration background N (%)	113	8		
Professional qualification				
Low^a^ N (%)	93	7		
Medium^b^ N (%)	597	43		
High^c^ N (%)	681	49		
Employment before delivery				
Not regularly employed N (%)	33	2		
Marginally employed N (%)	33	2		
Part-time employed N (%)	331	24		
Full-time employed N (%)	852	61		
Single parent N (%)	18	1		
Living space* (m^2^) (median, IQR)			120	(90–160)
Household size (number of persons) (median, IQR)			3	(3–4)
Infant				
Birth order				
First child N (%)	892	64		
Second child N (%)	393	28		
Third child or higher N (%)	107	8		
Gestational age at birth (weeks, Mean ± SD)			39,3	(1,6)

^a^Secondary/elementary education. ^b^Intermediate education. ^c^University entrance level/tertiary education.

*Total living space in the household.

### Infant sleeping location

**Figure 1:**
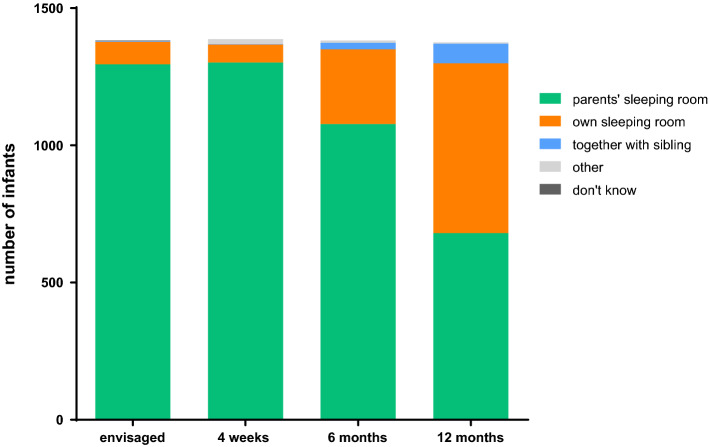
Default sleep location (“*In which room does your child usually sleep at night?*”).

Immediately after birth, the overwhelming majority (94%) of mothers planned for their child to sleep in the parents’ sleeping room (n = 1,295) while 82 mothers intended an own sleeping room for the child (6%) (Fig. 1). The next survey at four weeks of age showed a virtually identical picture. During the first year of life, we observed a gradual increase in the proportion of children usually sleeping in an own bedroom, from 20% at 6 months (n = 273) to 45% at one year (n = 619). Only a tiny proportion of children usually slept in the same room with a sibling, reaching a percentage of 5% (n = 71) at 12 months of age.

### Sleeping furniture and bed-sharing

**Figure 2:**
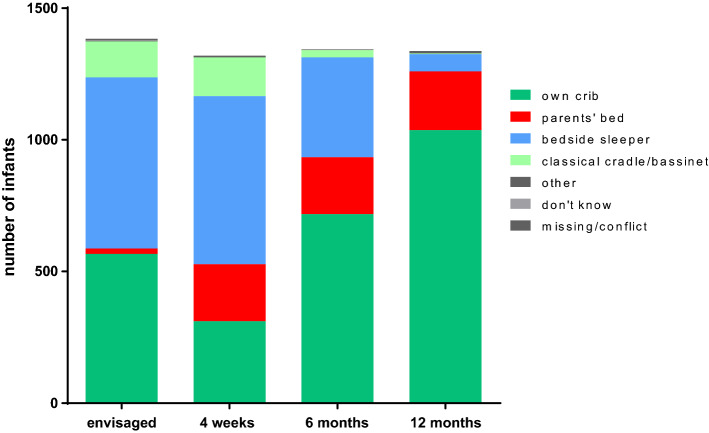
Standard sleeping furniture (“*What does your child usually sleep in at night?*).

Regarding the preferred bedroom furniture, we found a dynamic image. Before initial discharge from maternity ward, the proportion of families who intended the use of a (one sided-open) bedside sleeper and a baby crib were roughly equal in terms of magnitude (n = 650 and n = 567; corresponding to 47% and 41%) (Fig. 2). However, while the use of the bedside sleeper was virtually identical at four weeks of age (n = 639; 48%), the fraction of infants who slept in an own baby crib as standard sleeping furniture dropped to 24%. In turn, we saw a relevant rise at bed-sharing as the preferred sleeping location at four weeks of age. While in the interview after delivery, only a minuscule proportion of 1% stated that they plan to practice bed-sharing (n = 20), in the resurvey at four weeks of age, 16% of the participating families stated that the parents’ bed was actually the default sleeping place for their infant (n = 216). This proportion of bed-sharing as the standard then remained constant throughout the first year of life (16%; n = 216 at 6 months and 17%; n = 223 at 12 months).

A further subanalysis of the migratory balance (intended immediately after birth vs. practiced with 4 weeks) between the different default sleeping furniture showed that bed-sharing was the only sleeping place with a positive migratory net balance against all other items (Supplemental Fig. 1). On the other hand, we observed a marked negative net balance, especially for the infant sleeping in his/her own bed. This means that a considerable proportion of infants whose parents’ originally intended the use of an own baby bed as the default sleeping place in the net balance “moved” to the other sleeping furnitures during the first month of life. From four weeks of age onwards, we observed a constant and gradual increase for the own crib while the proportions of bedside sleeper and classical cradle/bassinet declined continuously. At the time of the first birthday (12 months), almost all children in our cohort study either slept in their own bed or co-slept together with an adult in the parents’ bed as the standard sleeping location.

**Figure 3:**
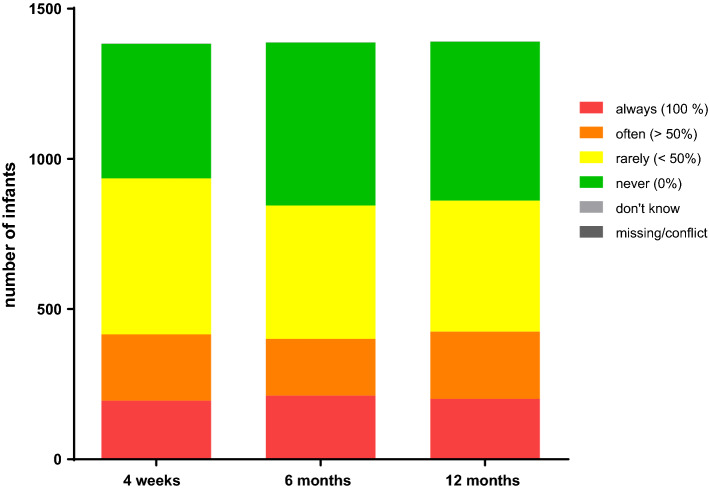
Frequency of bed-sharing (“*How often does your infant co-sleep with an adult in one bed at the same time?*”).

As bed-sharing with an adult displays an important risk factor for SIDS^11^, we further analyzed and quantified the bed-sharing practice in our cohort, even if practiced part-time and not as the main or exclusive sleeping location (Fig. 3). Only about one-third of the surveyed families never practiced bed-sharing with the infant at all and another third of families practiced bed-sharing sometimes. After the age of four weeks, the overall pattern of bed-sharing frequency remained quite static with no major shifts during the first year of life.

### Sleeping position

**Figure 4:**
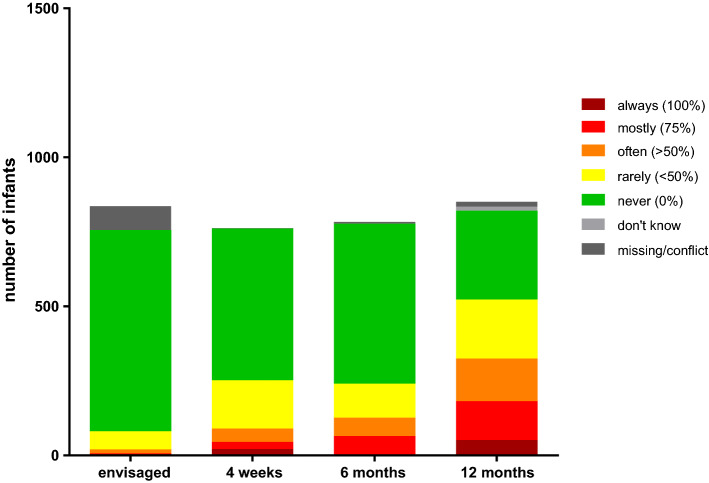
Frequency of prone positioning for sleeping (“*How often do you put your baby in the prone position ("face-down") for sleeping?*”).

In the interview after delivery, the vast majority of mothers stated that they would never actively put their infant in the prone position (i.e. face-down) for sleeping (Fig. 4). While this group remained the largest share throughout the first year, we observed a gradual rise of children who were put sometimes, often, or even mostly in the prone position for sleeping. Already at the earliest point in time, 2% of the interviewed mothers reported that they plan to put the infant to sleep in a face-down position most of the time. At the time of the first birthday, a diverse picture emerged with relevant proportions of all groups.

### Loose objects in the infant’s bed

**Figure 5:**
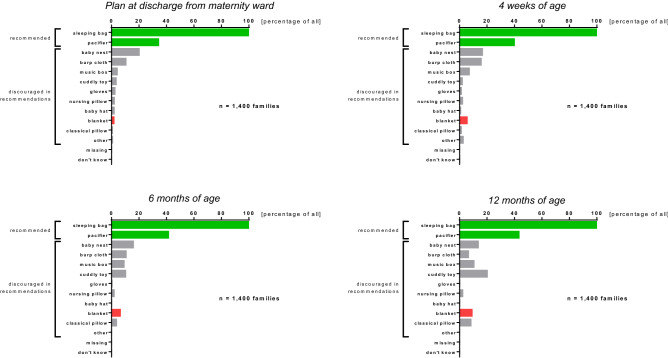
Loose objects in the infant’s bed (*“Which of the following objects do you give the infant into the bed for sleeping?”*).

To reduce the risk of suffocation, the current SIDS guidelines recommend that the infant is placed in a sleeping bag instead of a loose blanket. While virtually every family in our cohort (> 97% of 1,400 families) used a sleeping bag for their child, an additional blanket was provided in 4–25% and a baby nest and/or a nursing pillow was placed inside the bed of 15–20% of the infants, depending on the time of the assessment (Fig. 5). A pacifier, also recommended by the guideline, was offered to approximately 40% of children.

### External and environmental factors for SIDS risk

The most frequently targeted room temperature for the infant’s sleeping room was 18–20 °C at all investigation time points and was reported by approximately 40–50% of the families (Supplemental Fig. 2). The recommended room temperature of 16–18 °C was stated as preferred by 36% in the post-delivery interview and implemented in 23% (four weeks) to 18% (one year) later during the first year of life. However, we would like to point out that this analysis does not correct for the season of the survey (and hence the outside temperature), but indicates which temperature was targeted by the parents.

Regardless of nutritional, allergological, psycho-emotional, and other important aspects, breastfeeding (or receiving expressed milk) has been shown to be protective against SIDS^12^. In our cohort, the vast majority of infants received breast milk up at some point during the first year of life while at 12 months, only 337/1400 mothers (24%) still breastfed their child (Supplemental Fig. 3). An important environmental risk factor for SIDS is the exposure to tobacco smoke. Therefore, we determined the number of people in the infant’s household who regularly smoke. While 1,141 infants (81%) were born into non-smoking families, 213 infants (15%) lived in a household with one smoker, and 21 infants (2%) in a household with two smokers (Data not shown).

### Association analysis of different risk factors

Breastfed infants were more likely to practice bed-sharing [181/799 (22.7%) vs. 20/359 (5.6%), *p* < 0.001] but less likely to live in a family with a smoker [95/799 (11.9%) vs. 70/359 (19.5%), *p* < 0.001]. There were no other significant differences between infants who were breastfed and those who were not breastfed with regards to their sleeping practices. We did not see an association of bed-sharing with prone positioning at any timepoint [prone positioning in the bed-sharing vs. not bed-sharing group; at 4 weeks: 34/415 (8.2%) vs. 54/967 (5.6%), *p* = 0.09; at 6 months: 42/401 (10.5%) vs. 128/986 (13.0%), *p* = 0.23; at 1 year: 94/425 (22.1%) vs. 230/965 (23.8%), *p* = 0.53]. Living in a household with a smoker was not associated with prone positioning [43/234 (18.4%) vs. 236/1141 (20.7%), *p* = 0.48].

## Discussion

The principal finding of our study is that the majority of the 1,400 surveyed families in our birth cohort generally followed pivotal recommendations for SIDS prevention during the infant’s first year of life. This was especially true for the recommendation that infants should sleep in their parents’ sleeping room and that a sleeping bag instead of a blanket should be used. However, we also observed a remarkable proportion of infants who were still placed regularly or even preferably in the prone position for sleeping. The most prevalent deviation from the relevant SIDS guideline in our current analysis was bed-sharing with an adult, which was practiced in a considerable part of the families. For every sixth infant, bed-sharing in the parent’s bed actually was the default sleeping location.

A large majority of parents is well informed regarding measures to prevent SIDS and consequently they mostly plan to abide by these recommendations^8^. In the current study, we could show that parents not only know but appear to largely follow the recommendations through the first year after birth. Notably, most infants initially slept in their parents’ bedroom and slowly moved to their own room during the first year. Also, it is encouraging to note that most infants were supplied with a sleeping bag instead of a loose blanket. This finding is in line with Shapiro-Mendoza who reported a marked trend against the use of blankets^13^. Regarding other loose items in the infant’s bed, the most frequently used was a baby’s nest and a nursing pillow (U-shaped pillow), which was put into the bed of approximately 20% of the infants. Although the exact attributable risk of such pillows is still unclear, several cases of sudden unexplained infant deaths cases have been reported in this context^14^ and the guidelines recommend against the use^1^. Generally, there should be no loose items at all in the bed that bear the risk of obstructing the infant’s breathing.

To the best of our knowledge there are hitherto no studies which determined the association between bedside sleepers and SIDS risk. Accordingly, there is no evidence for a clear recommendation and hence the SIDS task force of the *American Academy of Pediatrics* recommends neither for nor against the use of bedside sleepers^1^. The national German guideline on SIDS prevention does not even mention bedside sleepers^4,5^. During the recruitment period for our birth cohort, an update of the corresponding German SIDS guideline has been issued^4^. However, regarding the parameters assessed in our present paper, there have been no relevant changes compared to the previous version^5^. At least, the US *Consumer Product Safety Commission* has issued safety standards for bedside sleepers^15^. As a bedside sleeper seems to become the most common bedroom furniture for infants (at least in our study region), further epidemiological studies on the impact on SIDS are urgently needed.

Unlike for bedside sleepers, data regarding bed-sharing and SIDS are available and several studies showed an increased SIDS risk for infants bed-sharing in an adult’s bed^16,17^, especially in the context of alcohol or drug use or in special situations, like sleeping on a sofa^18^. Consequently, in most (but not all) current guidelines in Western countries, bed-sharing with an adult is discouraged, i.a. in the German guideline^4^, which is relevant for our study collective. However, this topic is subject of the ongoing scientific discussion and other authors emphasize further important aspects of bed-sharing^19^, e.g. facilitation of breastfeeding, bonding aspects, or improvement of sleep quality for the infant-parent tryad. Some authors even no longer regard bed-sharing as a modifiable risk factor in its proper sense which can be influenced by simple recommendations^20^ and suggest a more balanced approach to parental counseling^21^*.* However, the aim of our present study was not to question the content of the guideline which is currently applicable to the study population, but to determine the actual implementation by the families.

In a previous study, we found that most parents are well aware that the current SIDS guideline discourages from bed-sharing with an adult and intend to follow the corresponding recommendation not to share the bed with the infant^8^. Immediately prior to initial discharge from maternity ward, only 2% of interviewed mothers indicated that their child would be regularly allowed to sleep in the parent's bed. However, our current analysis of the actual implementation illustrates a considerable gap between intentions and real life behavior with only about one-third of the surveyed families never practicing bed-sharing with the infant at all. These observations are in line with Stromberg et al. who reported that bed-sharing was frequently practiced and that up to 25% of infants under three months almost exclusively slept in the parental bed^22^. In our current study, overall two thirds of infants co-slept in their parents' bed at least some of the time. Considering a potential social desirability bias, this rate may well be higher. The subanalysis of the migration balance sheds light on an interesting aspect in this context: while bed-sharing was the only sleeping furniture with a positive net balance against all other items, in summary we observed a particularly pronounced net migration from “own infant’s bed” to “bed-sharing” between birth and four weeks of age. Although a survey of parents' motivations for this behavior was not part of our present study, other authors analysed the reasons for bedsharing^19^ and showed that a major point was facilitation of nighttime care and breastfeeding. Whether the usage of a bedside sleeper will bring a change in this respect and might lead to a more stable avoidance of bed-sharing can only be speculated about and should be addressed in a future study.

In summary, we have to acknowledge that the majority of the parents in our sample (and in many others) are unwilling to follow the current guidelines regarding bed-sharing. An assessment of the possible reasons for this deviation from official advice was not part of our study, but at least the significant association between bed-sharing and breastfeeding suggests an important partial explanation. In general, when condering our data on bed-sharing, it is important to realize that our study sample does not represent a high-risk collective for SIDS, such as low-birth weight or premature infants. It will be subject of the scientific debate, whether future recommendations for parents in such a collective should be less categorically against bed-sharing but modified towards a risk-graded recommendation. Considering that two-thirds of families in our survey obviously did not follow the blanket recommendation not to bed-share, it appears that the current practice of counseling is not working. Whether a more targeted approach, focusing on specific high-risk infants, might be overall more effective needs to be clarified in future studies. Regardless of this thought for future advancement of SIDS counseling, all parents should be educated by the health care professionals about the potential benefits and the risks of bedsharing.

In the *National Infant Sleep Position Study*, Colson and colleagues showed that between the years 1993 and 2000 the percentage of parents who exclusively placed their child on the back was continuously rising^23^. However, since 2001 this trend has reached a plateau. While the majority of mothers in our study had the intention to never actively put their infant in the prone position for sleeping, the number of infants being actually placed in prone position increased over the first year of life. Already at the age of four weeks, approximately one third of the surveyed infants was placed in a prone position sometimes or more often. However, the occasional prone position is of particular concern, as infants who are not accustomed to this position carry a particularly high risk of SIDS^24^. As most parents were aware of SIDS prevention measures after birth^8^, but their good intentions were not consistently implemented during the first year of life, we consider that these recommendations need to be addressed repeatedly to increase the level of actual implementation.

Among the environmental factors for SIDS, exposure to tobacco smoke is an important modifiable risk factor^25–27^. An analysis of a large longitudinal cohort from 2012 found that 43% of German children under 17 years have at least one parent who smokes^28^. In our cohort, almost a quarter of the children were born into households with a smoker, reflecting a significant number of infants who are still going to be exposed to this avoidable risk factor for SIDS. Moreover, we have to realize that questions regarding parental smoking behavior are especially prone to social desirability bias. Thus, the actual number may even be higher than reported by the parents in this study.

Our study has some limitations. First, there is a potential selection bias as parents had to have sufficient German language skills to be included. Since ethnic differences can impact sleeping habits in families^29–31^, studies in different cultural collectives may yield different results. Furthermore, parents with higher educational status are overrepresented in the KUNO-Kids birth cohort^9^. This circumstance led to a difference in some socio-economic characteristics between the drop-outs and the families that remained in the cohort. Specifically, families with only one child, an older age of the mother, a mother with higher education level, a mother with full-time employment before birth of the child, and a mother without a migration background were more likely to continue participating in the study (for details see Brandstetter et al.^9^). Therefore when interpreting our data, it is important to keep in mind that, conversely, families with lower socio-economic status and/or with a migration background are significantly underrepresented. In the context of SIDS prevention this aspect has to be considered, as there seems to be an association between SIDS risk behavior and low socio-economic status^16,32^. Another limitation of our analysis is that we included primiparae as well as multiparae. However, for mothers with already one older child (or several older children as well), the prior exposure to SIDS recommendations and the experience with their implementation is likely to influence the implementation with the current child, particularly if the mother attempted to comply with guidance previously and found it difficult to do so. A further point, which has to be considered when interpreting our data, is that infants very likely might have moved from one place to the other during the night. Nevertheless, in order to keep the questionnaires as simple as possible for the parents and thus to achieve a better response quality, in this survey we focused on asking about the default primary sleeping place where the baby slept most of the time. Therefore, we might have underestimated the role of some secondary sleeping places (especially during daytime), for example the frequency of bed-sharing. Moreover, as the KUNO-Kids birth cohort is an interdisciplinary study covering multiple domains of pediatric health and development, we were forced to limit data collection in our substudy to an appropriate number of items. Hence, we focused on key aspects of SIDS prevention but were unable to assess some detailed aspects that might also have been of interest, such as the differentiation between nighttime and daytime sleep or the combination of different risks, such as bed-sharing plus smoking or alcohol consumption. Finally, there was a notable dropout of families from the baseline interview after birth to the one-year questionnaire. This may have further selected the most conscientious families who may be more prone to follow recommendations. In summary, the actual situation with respect to adherence to SIDS prevention recommendations in the first year of life might be even worse than reported in this study.

## Conclusion

In conclusion, although most parents in our current survey implemented many SIDS recommendations, we observed a remarkable proportion of infants who were still routinely placed in the prone position for sleep or who were given loose objects into the bed, e.g. a baby nest or a nursing pillow. The number-one deviation from the current SIDS guidelines during night-time sleep was bed-sharing with an adult, which was practiced in a substantial number of families and was the default sleeping location for every sixth infant.

## Supplementary Information

Supplementary Figures.

## Data Availability

The datasets analysed in the current study are available from the corresponding author on request.
