# Supplementary material for: Implementation of safe infant sleep recommendations during night-time sleep in the first year of life in a German birth cohort
Source: Sci Rep. 2023 Jan 17;13:875. doi: 10.1038/s41598-023-28008-1 (PMC9845375; doi:10.1038/s41598-023-28008-1)
Supplement: Supplementary file 1 — Supplementary Figures. [file 41598_2023_28008_MOESM1_ESM.docx]

**Supplemental Figures to**

**Implementation of safe infant sleep recommendations during night-time sleep in the first year of life in a German birth cohort**

**Vincent D. Gaertner ^1, 2^ Sara Fill Malfertheiner ^3^, Janina Postpischil ^1^, Susanne Brandstetter ^4, 6^ Birgit Seelbach-Göbel ^3^, Christian Apfelbacher ^4, 5^, Michael Melter ^6^, Michael Kabesch ^1, 4^, and Sebastian Kerzel* ^1^ and the KUNO-Kids study group**

^1^ Department of Pediatric Pneumology and Allergy, University Children’s Hospital Regensburg (KUNO) at the Hospital St. Hedwig of the Order of St. John, University of Regensburg, Germany

^2^ Dr. von Hauner Children's Hospital, University Hospital, Ludwig-Maximilians-University Munich, Munich, Germany.

^3^ University Department of Obstetrics and Gynecology at the Hospital St. Hedwig of the Order of St. John, University of Regensburg, Germany

^4^ WECARE Research and Development Campus Regensburg at the Hospital St. Hedwig of the Order of St. John, Regensburg, Germany

^5^ Institute of Social Medicine and Health Systems Research (ISMHSR), Otto von Guericke University Magdeburg, Magdeburg, Germany

^6^ University Children’s Hospital Regensburg (KUNO), University of Regensburg, Germany.

**Supplemental Figure 1: Migratory balance of the infant’s sleeping furniture
(birth vs. 4 weeks)**

**A**

**
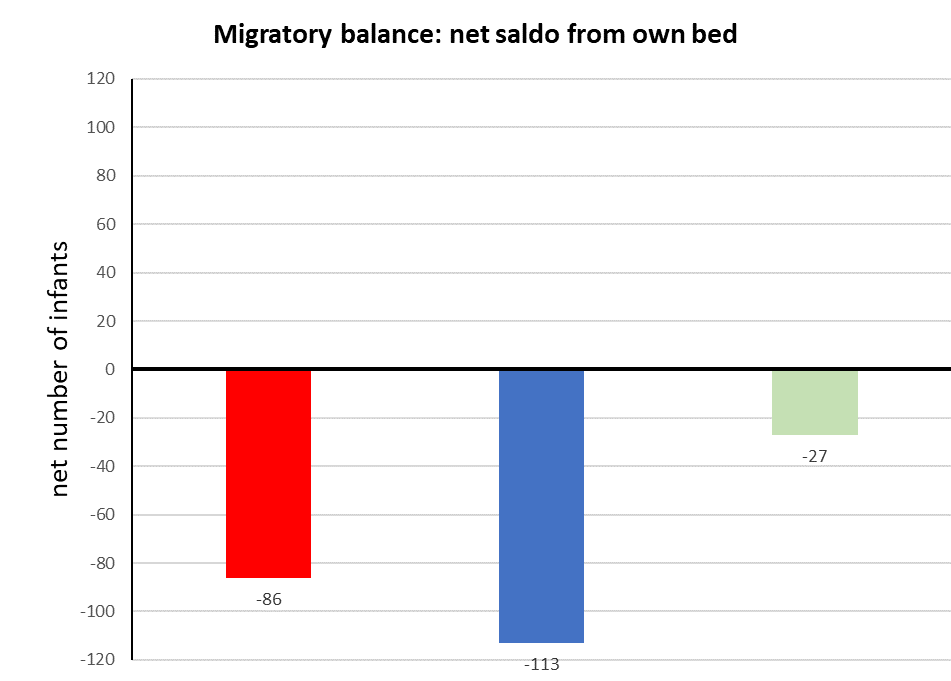
**

to bedside sleeper

to bedsharing

to cradle

**B**

**
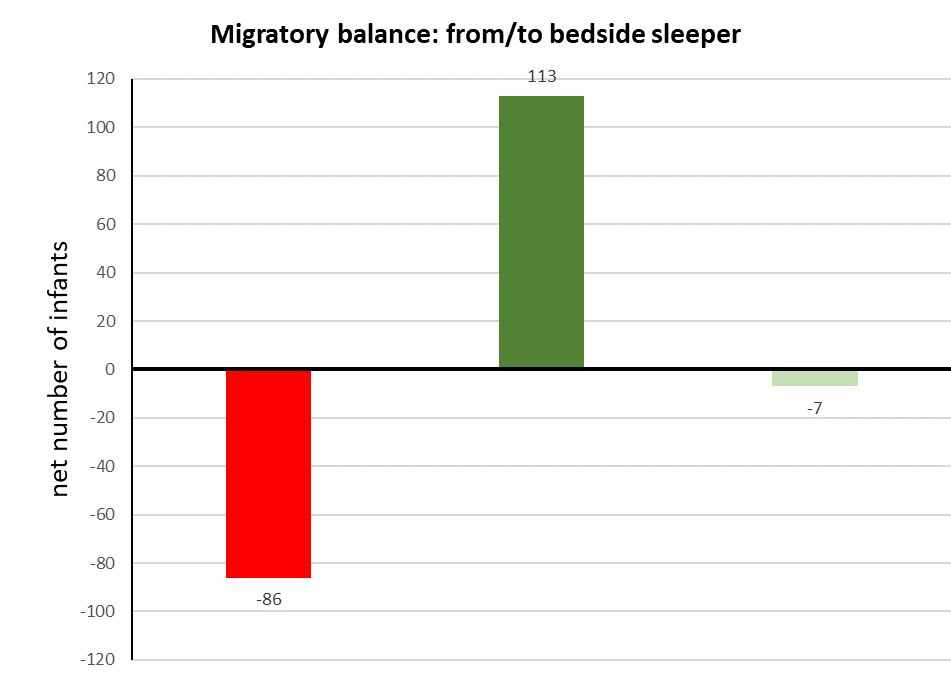
**

from own bed

to cradle

to bedsharing

**C**

**
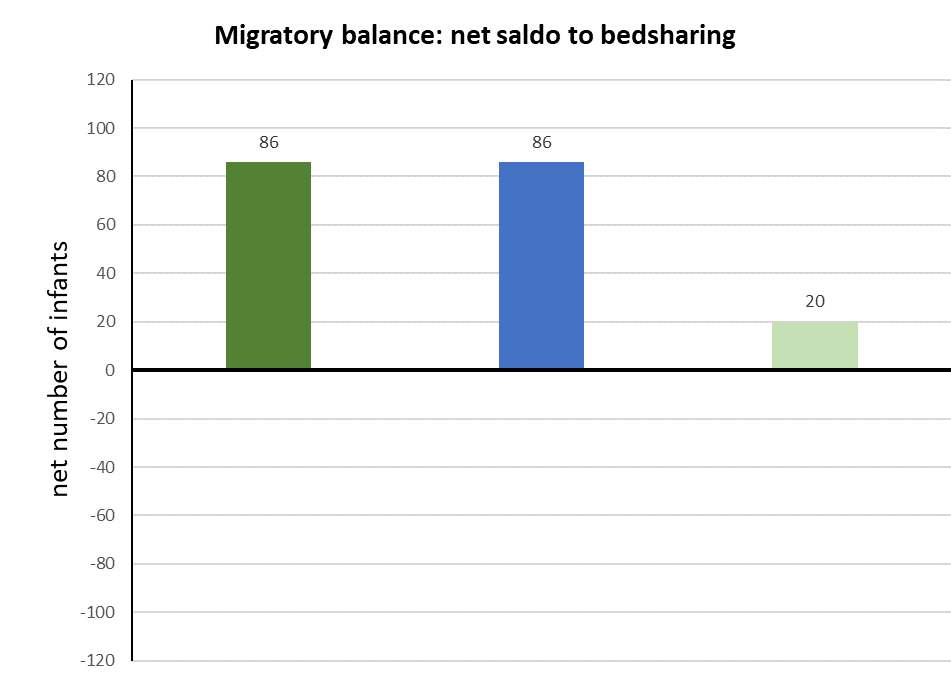
**

from bedside sleeper

from own bed

from cradle

**Supplemental Figure 2: Sleeping room’s temperature**

**Supplemental Figure 3: Nutrition with breastmilk**
